# Animal Models of Human Pathology 2014

**DOI:** 10.1155/2015/721348

**Published:** 2015-07-12

**Authors:** Monica Fedele, Oreste Gualillo, Andrea Vecchione

**Affiliations:** ^1^Istituto per l'Endocrinologia e l'Oncologia Sperimentale (IEOS), CNR, 80131 Naples, Italy; ^2^Santiago University Clinical Hospital, Instituto de Investigación Sanitaria de Santiago (IDIS), 15706 Santiago de Compostela, Spain; ^3^Department of Clinical and Molecular Medicine, Sapienza University of Rome, 00100 Rome, Italy

In this special issue we have assembled original research as well as review articles describing researches in which animal models, ranging from small vertebrates to larger animals, have been used for either understanding the molecular events underlying a disease or improving therapy in various fields of medicine. Despite the widespread use of* in vitro* systems that allow recapitulating almost all the processes involved in the development of a disease, only the use of animal models enable us to reproduce onset mechanisms of human disease, thereby providing the only system known today to test new drugs for therapeutic interventions. Last year has been an exciting year for animal models. Indeed one of the most extraordinary discoveries of the last years, which has been largely implemented in 2014 transforming and expanding our ability to model human diseases in animals, has been the CRISPR- (clustered regularly interspaced short palindromic repeat-) Cas9 (CRISPR-associated nuclease 9) system, an easy method to efficiently manipulate the genome of any organism [1, 2]. This gene editing approach is based on a RNA-guided nuclease (Cas9) that generates targeted double-stranded DNA breaks and coopts the endogenous cellular pathways to repair them while introducing precise changes into the genome. The specificity of the genomic locus where Cas9 is recruited is driven by a small guide RNA (gRNA) that is complementary to the target site. The CRISPR-Cas9 system is a revolutionary technology that is being applied in a wide variety of organisms, including mice, rats, zebrafish, monkeys, and butterflies [3–7]. One of the best explored human pathologies using this method has been cancer. Two sister studies published in December 2014 on Nature efficiently approached the CRISPR/Cas9 system to induce specific mutations in mice. The group of A. Ventura developed a new mouse model of Eml4-Alk-driven lung cancer by inducing the specific chromosomal rearrangement that leads to the generation of the EML4-ALK oncogene. The resulting tumors display histopathological and molecular features typical of ALK(+) human non-small cell lung cancer (NSCLC) and respond to treatment with ALK inhibitors [8]; using a well-established mouse model of lung cancer, T. Jacks group functionally characterized a panel of candidate tumor suppressor genes by inducing specific knockout mutations by the CRISPR/Cas9 method [9].

On the side of preclinical studies for drug discovery, the 2014 year has been also characterized by a surge in the numbers of studies employing the zebrafish model to discover and test new anticancer drugs. Indeed, in an era of crisis like that one, we are going through chemical screens in zebrafish result much more rapid and less expensive than the current preclinical studies, which are often bulky and costly, thus limiting the number of new drugs that can efficiently proceed to be used as therapy [10]. The zebrafish is a powerful model system for studying human cancer because of the ease with which it can be genetically manipulated, and the opportunity to be directly observed in transparence. Therefore, several mutant and transgenic zebrafish have been generated to model human cancers and have been used for* in vivo* drug screenings. In particular, xenotransplantation of human cancer cells into zebrafish embryos enables a direct* in vivo* evaluation of patient-derived tumor material in a cost-effective and time-efficient manner.

We hope that this special issue will serve to all the readers as valuable source of scientific background and inspiration in this exciting field of research.
